# The directed acyclic graph helped identify confounders in the association between coronary heart disease and pesticide exposure among greenhouse vegetable farmers

**DOI:** 10.1097/MD.0000000000035073

**Published:** 2023-09-22

**Authors:** Honghui Li, Cheng Zheng, Yue Zhang, Huifang Yang, Jiangping Li

**Affiliations:** a Department of Occupational and Environmental Health, School of Public Health and Management, Ningxia Medical University, Yinchuan, Ningxia Hui Autonomous Region, China; b Department of Epidemiology and Statistics, School of Public Health and Management, Ningxia Medical University, Yinchuan, Ningxia Hui Autonomous Region, China.

**Keywords:** binary logistic regression, coronary heart disease, directed acyclic graphs, pesticide exposure

## Abstract

To explore the causal pathways associated with coronary heart disease (CHD) and pesticide exposure using a directed acyclic graph (DAG) analysis and to investigate the potential benefits of DAG by comparing it with logistic regression. This cross-sectional study enrolled 1368 participants from April 2015 to May 2017. Trained research investigators interviewed farmers using a self-administered questionnaire. Logistic regression and DAG models were used to identify the associations between CHD and chronic pesticide exposure. A total of 150 (11.0%) of the 1368 participants are characterized as having CHD. High pesticide exposure (odds ratio = 2.852, 95% confidence intervals: 1.951–4.171) is associated with CHD when compare with low pesticide exposure by both DAG and logistic analyses. After adjusting for the additional potential influence of factors identified by the DAG analysis, there is no significant association, such as the results in logistic regression: ethnicity, education level, settlement time, and mixed pesticide status. Specifically, age, meal frequency, and consumption of fresh fruit, according to the DAG analysis, are independent factors for CHD. High pesticide exposure is a risk factor for CHD as indicated by both DAG and logistic regression analyses. DAG can be a preferable improvement over traditional regression methods to identify sources of bias and causal inference in observational studies, especially for complex research questions.

## 1. Introduction

Coronary heart disease (CHD) remains the leading cause of death worldwide with approximately 17.8 million people killed by cardiovascular disease around the world, and 8.9 million of these died of CHD in 2017.^[1,2]^ Although there are many contributing causes and their associated risk factors (e.g., type 2 diabetes mellitus, hypercholesterolemia, hypertension, and psychological problems) to CHD, exposure to pesticides is associated with increased mortality and may have some effects on the cardiovascular system.^[3–5]^

Pesticides are unique environmental perturbations that are specifically introduced into agriculture to control pests by killing them. It is worth noting that global pesticide production grew at an annual rate of about 11% per year, from 0.2 million tons in the 1950s to more than 5 million tons by the 2000s.^[6]^ Three billion kilos of pesticides per year are used worldwide,^[7]^ but only 1% of total pesticides employed are effectively used to control insect pests on target plants.^[8]^ For example, in China, the chemical pesticides used per unit area of arable land is nearly 2.5 to 5 times the world average.^[9]^ A recent study points out that pesticides are overused in the greenhouse farming of vegetables and fruits, and levels of pesticide residues in greenhouses were much higher than for open-field vegetables.^[10,11]^ However, more than 90% of China’s fresh vegetables and fruits are planted in greenhouses,^[8]^ and that food may account for most of the pesticide use in China. Thus, the health effects of long-term pesticide exposure have become increasingly relevant as the number of greenhouse farmers increases.

Previous studies have proven a positive linkage between pesticide exposure and CHD,^[12]^ but most previous studies on occupational pesticide exposure and CHD only focused on mortality due to disease,^[13,14]^ and the causal pathways are not yet fully understood. The influencing factors for CHD are numerous and closely related, which impedes the establishment of causal relationships between pesticide exposure and CHD risk, and confounding factors are difficult to control.

There is uncertainty as to which factors affecting pesticide exposure and CHD are confounding factors. In general, confounding factors are covariates that are associated with, but not affected by, exposure, and are directly responsible for the outcome. A multivariate regression can be adjusted for potential confounding variables as a simple method of reducing confounding bias. However, this conventional approach may produce other biases, such as collider bias,^[15]^ rather than reducing bias. Furthermore, the traditional gold standard for investigating causality in randomized trials. However, most questions concerning the causal mechanisms of illness cannot be studied in randomized trials.^[16]^ To make effective causal inferences from observed data, it is necessary to determine appropriate methods to control confounding factors. However, confounding factors are not always easy to recognize when using traditional multiple logistic regression.^[17,18]^ One recently exploited approach for recognizing confounding factors is the use of directed acyclic graphs (DAG), which are visual representations of causal assumptions that are being increasingly used in contemporary epidemiology practice.

This study aimed to adjust binary regression models for the sets of covariates to allow estimation of the relationship between CHD and pesticide exposure in a less biased manner, by drawing a DAG to identify and control for potential confounding factors. We wanted to investigate the potential benefits of DAG by comparing it to logistic regression and causal inference for risk prediction.

## 2. Material and methods

### 2.1. Study design and participants

This cross-sectional study enrolled a total of 1368 vegetable greenhouse farmers (448 participants were interviewed in 2015, 460 participants in 2016, and 458 participants in 2017) consecutively recruited from April 2015 to May 2017, from 4 villages (Liangtian, Maosheng, Yinhe, and Wudu) in the suburbs of Yinchuan. Yinchuan, the capital of Ningxia Province in Northwestern China, is the most important farming center in Ningxia. This study was intended to collect locally representative specimens to determine the relationship between the health of greenhouse farmers and pesticide exposure status. Non-repeated residents were randomly selected from 4 rural areas per year, and all who met the requirements were invited to participate in the investigation. Five hundred self-administered questionnaires to each village were distributed every year. The response rates were over 70% for each cycle.

The inclusion criteria for all participants were: (1) residents over the age of 18; (2) farmers who lived at their current address for more than 5 years and were engaged in greenhouse vegetable planting for at least 1 year. The exclusion criteria were: (1) residents who do not agree to participate or the questionnaire has not been completed in this study; (2) discontinuous greenhouse growers; (3) with previous or family history of CHD. For all participants with CHD who recorded such a diagnosis, we requested and reviewed medical records.

These data were collected by the researchers during greenhouse work, often with verbal consent from the participating farmers or paraphrased with the help of a family member. The study was approved by the Medical Ethics Committee of Ningxia Medical University (No. 2014-090) and performed by the declaration of Helsinki. All greenhouse practitioners or their legal representatives willingly completed and signed the informed consent form.

### 2.2. Data collection procedure

The questionnaire mainly included sociodemographic information (age, sex, residence time, marital status, etc), socioeconomic information (education level, household income, etc), health outcomes at enrollment (diabetes, hypertension, etc), and other conditions that might impact health (diet, smoking consumption, alcohol consumption, local fruit and vegetable consumption, and pesticide exposure). The smoking status was classified into current (smoking more than 1 cigarette per day, continuous or accumulative 6 months), occasional or former (smoking cigarettes more than 4 times a week, but <1 cigarette per day on average), and never.^[19]^ Alcohol drinking was classified as never, 30 days ago, within 30 days (at least once 2 weeks).^[20]^ Included were patients diagnosed at county-level hospitals or above after clinical examination.

### 2.3. Pesticide exposure intensity

Greenhouse pesticide exposures are grouped by the score for personal protective equipment (PPE), as indicated by the answer to the following question: “What safeguards do you take when spraying pesticides?” If “none” was chosen, the practitioner was classified as PPE-0. If one or more of the following options were chosen, the practitioner was classified as PPE-1: protective masks, goggles, fabric or leather gloves, and worn-out clothes. If one or more of the following options was chosen, the practitioner was classified as PPE-2: gas masks, protective rubber boots, or a protective suit. If rubber gloves were reused, then the practitioner was classified as PPE-3. The PPE scores are shown in Table S1, Supplemental Digital Content, http://links.lww.com/MD/J779. A higher score indicates a lack of personal protection awareness during pesticide spraying. Risky behaviors during pesticide spraying were divided into 5 categories: eating, drinking water or beverages, smoking, calling or chatting, and none; scores were 3, 3, 2, 1, and 0, respectively.

### 2.4. DAG methods

DAG, which provides a systematic representation of causal associations, has become established as a relatively new framework for the analysis of causal inference in epidemiologic research.^[15,16]^ DAG is a type of causal diagram comprising nodes representing variables, and arrows representing causal relationships between the variables. Regardless of direction, any series of adjacent arrows in the diagram will form a path. The series must be acyclic, which means that it cannot form a feedback loop in which a path from a variable causes itself. DAG is closely related to the more familiar path diagrams but differs in that path diagrams also imply a parametric linear statistical relationship between variables.^[21]^ The PC algorithm proposed by Spirtes et al determines the DAG through 2 stages: elimination and orientation.^[22]^ In the elimination stage, the insignificant edges are first removed by testing the significance of the unconditional correlation coefficient between variables, and then the remaining edges are removed. In the orientation stage, the number of partial correlation coefficient tests is the number of variables minus 2. The condition variable when the undirected edge between variables is eliminated in the test process is called the separation set between variables; in the orientation stage, there is a criterion to determine the causal relationship.

### 2.5. Definition of covariates

To facilitate the manipulation of arrows for causal interpretation, we imposed the following knowledge-based constraints: In greenhouse operations, personal protection awareness can directly affect pesticide spraying behavior, which in turn influences pesticide exposure, and both factors can directly cause pesticide exposure.^[23]^ Pesticide exposure, the crucial factor affecting health, can directly affect the health of individuals. A study demonstrated that there is a direct correlation between CHD and the intensity of pesticide exposure.^[12]^ Therefore, CHD was specified as a sink with only inward-pointing arrows, and pesticide exposure was specified as a source with only outward-pointing arrows in the current study. Moreover, many controllable factors such as smoking,^[24]^ drinking,^[25]^ physical activity,^[26]^ the intake of fresh fruits and vegetables,^[27]^ and a diet high in salt 5 are related to CHD development. Other risk factors include many nonmodifiable variables, such as age,^[24]^ sex,^[28]^ and ethnicity^[29]^; those factors have previously been demonstrated to be directly associated with CHD. Additionally, established evidence indicates that income status plays a vital role in affecting exposure in greenhouses, and is directly connected with smoking, alcohol consumption, exercise, the consumption of meat, fresh vegetables, and fruits, etc.^[30]^ A larger number of family members will not only directly reduce the hours of work in the shed per practitioner, but will also alleviate the labor burden of family members, which may reduce the intensity of an individual worker’s shed exposure. Education level is of great importance to allow a worker to recognize pesticide exposure and take effective protective measures.^[31,32]^ Hence, family members and education level indirectly affect the occurrence of CHD by affecting pesticide exposure. The Tetrad software was applied with these constraints to valid survey data and the DAG model was automatically generated.

### 2.6. Statistical analysis

Initial differences in the distributions of the baseline characteristics, such as demographic characteristics, lifestyle characteristics, and the greenhouse planting situation were assessed with the *χ*2 test or the Fisher exact test, as appropriate. To investigate factors affecting CHD, a logistic regression model was used to evaluate the relationship between hazard ratios for CHD and numerous covariates. Relative risks were estimated by calculating the odds ratio (OR) and 95% confidence intervals (CIs). Next, a DAG was drawn based on a causal path between potential covariates, exposure, and outcome variables. All statistical tests were performed with SPSS software version 23.0 (SPSS, Inc., Microsoft, Chicago, IL). A *P*-value threshold of .05 was considered statistically significant. The DAG was drawn by using tetrad 6.9.0 web-based software (Tools—Center for Causal Discovery (pitt.edu)).

## 3. Results

### 3.1. Baseline characteristics of patients

Table 1 compares baseline CHD risk factors in participants who developed incident CHD during the investigation with those who remained free of CHD. There were 1366 participants in this study (448 participants were interviewed in 2015, 460 participants in 2016, and 458 participants in 2017), 726 males and 642 females. A total of 150 (11.0%) participants were characterized as having CHD. The mean age was 46.0 years (SD 10.5 years). Significant differences in incident CHD occurred for age, ethnicity, household expenses, consumption of sauerkraut, behavior, mixed pesticide status, pesticide spraying frequency, and degree of exposure. Age over 45 years, high household expenses, edible sauerkraut, 2 or more mixed pesticides, a high pesticide spraying frequency, and a high pesticide exposure had a higher risk of incident CHD (*P* < .05). Details are shown in Table 1.

**Table 1:** Baseline cardiovascular risk characteristics stratified by disease status.

Characteristics	Overall (n, %)	CHD (n, %)	No CHD (n, %)	*P*-value
*Demographic characteristics*				
Family members				.137
≤2	138 (10.1)	12 (8.0)	124 (10.3)	
3	235 (17.3)	34 (22.7)	198 (16.4)	
≥4	987 (72.6)	104 (69.3)	882 (73.3)	
Age (years)				.003
≤45	617 (47.1)	90 (60.0)	523 (45.2)	
45–60	553 (42.2)	47 (31.3)	505 (43.7)	
>60	141 (10.8)	13 (8.7)	128 (11.1)	
Sex				.386
Male	726 (53.1)	85 (56.7)	638 (52.6)	
Female	642 (46.9)	65 (43.3)	574 (47.4)	
Ethnicity				<.001
Han	1213 (88.7)	118 (78.7)	1090 (89.9)	
Hui	153 (11.2)	32 (21.3)	122 (10.1)	
Marital status				.676
Unmarried	42 (3.0)	6 (4.0)	36 (3.0)	
Married	1298 (95.0)	142 (94.7)	1151 (95.0)	
Divorced	27 (2.0)	2 (1.3)	25 (2.1)	
Education level				.072
No formal schooling	374 (27.4)	28 (18.7)	345 (28.5)	
Primary school	437 (32.0)	54 (36.0)	381 (31.4)	
Middle school	466 (34.1)	55 (36.7)	409 (33.7)	
Secondary school and higher	90 (6.6)			
Household income				.216
<4000	50 (3.9)	2 (1.3)	48 (4.2)	
4000–10,000	346 (36.7)	44 (29.3)	301 (26.3)	
10,000–20,000	381 (29.4)	49 (32.7)	331 (29.0)	
>20,000	521 (40.1)	55 (36.7)	463 (40.5)	
Household expenses				.014
<4000	221 (17.8)	22 (15.1)	199 (18.3)	
4000–10,000	624 (50.4)	83 (56.8)	538 (49.4)	
10,000–20,000	223 (18.0)	32 (21.9)	190 (17.4)	
>20,000	171 (13.8)	9 (6.2)	162 (14.9)	
Residence time				.056
<10 years	715 (52.4)	89 (59.7)	623 (51.4)	
≥10 years	650 (47.6)	60 (40.3)	588 (48.6)	
*Lifestyle*				
Smoking status				.753
Current	491 (35.9)	58 (38.7)	431 (35.6)	
Occasional/former	84 (6.1)	9 (6.0)	75 (6.2)	
Never	791 (57.9)	83 (55.3)	706 (58.3)	
Secondhand smoke (days/week)				<.001
7	558 (48.1)	50 (35.2)	508 (50.1)	
4–6	175 (15.1)	39 (27.5)	136 (13.4)	
1–3	30 (2.6)	3 (2.1)	27 (2.7)	
Never	396 (34.2)	50 (35.2)	343 (33.8)	
Alcohol drinking				.052
30 days ago	210 (15.4)	13 (8.7)	197 (16.3)	
Within 30 days	298 (21.8)	35 (23.3)	260 (21.5)	
Never	857 (62.8)	102 (68.0)	754 (62.3)	
Disease history				.162
No	1105 (80.9)	115 (76.7)	986 (81.4)	
Yes	261 (19.1)	35 (23.3)	225 (18.6)	
Physical activity				.665
No	12,122 (84.0)	127 (85.2)	992 (83.9)	
Yes	214 (16.0)	22 (14.8)	191 (16.1)	
*Diet*				
Meal frequency				.441
Once	18 (1.3)	2 (1.3)	16 (1.3)	
Twice	534 (39.1)	66 (44.0)	468 (38.6)	
Three times	809 (59.4)	82 (54.7)	727 (60.0)	
Meat				.075
No	41 (3.0)	1 (0.7)	40 (3.3)	
Yes	1324 (97.0)	149 (99.3)	1171 (96.7)	
Fresh fruits				.459
No	58 (4.3)	8 (5.3)	49 (4.0)	
Yes	1306 (95.7)	142 (94.7)	1161 (96.0)	
Fresh vegetables				
No	14 (1.0)	1 (0.7)	13 (1.1)	
Yes	1351 (99.0)	149 (99.3)	1198 (98.9)	
Sauerkraut				.009
No	487 (35.7)	68 (45.3)	418 (34.5)	
Yes	877 (64.3)	82 (54.7)	792 (65.5)	
Amount of salt				.484
Light	566 (41.4)	67 (44.7)	495 (40.8)	
Moderate	449 (32.9)	43 (28.7)	406 (33.5)	
Heavy	351 (25.7)	40 (26.7)	311 (25.7)	
*Greenhouse planting situation*				
Working hours (days)				.755
≤200	244 (18.0)	29 (19.5)	214 (17.8)	
200–300	366 (27.0)	42 (28.2)	322 (26.7)	
>300	748 (55.1)	78 (52.3)	669 (55.5)	
Mixed pesticides				.050
Current	600 (47.6)	118 (79.2)	840 (69.9)	
Occasional	396 (31.4)	14 (9.4)	190 (15.8)	
Never	265 (21.0)	17 (11.4)	171 (14.2)	
Mixing several pesticides (types)				.132
2	471 (44.6)	57 (51.4)	413 (43.8)	
≥3	585 (55.4)	54 (48.6)	529 (56.2)	
Pesticide spraying frequency (days)				.017
≤7	807 (62.2)	106 (72.6)	701 (61.1)	
7–14	299 (23.1)	28 (19.2)	269 (23.5)	
>14	191 (14.7)	12 (8.2)	177 (15.4)	
Pesticide spraying time (hours)				.529
≤1	782 (60.3)	91 (62.8)	688 (60.0)	
1–2	402 (31.0)	45 (31.0)	356 (31.0)	
>2	112 (8.6)	9 (6.2)	103 (9.0)	
Degree of exposure				<.001
Low exposure	424 (31.3)	82 (54.7)	342 (28.2)	
High exposure	938 (68.9)	68 (45.3)	870 (71.8)	

CHD = coronary heart disease.

### 3.2. Logistic regression model on CHD

**Figure 1.**
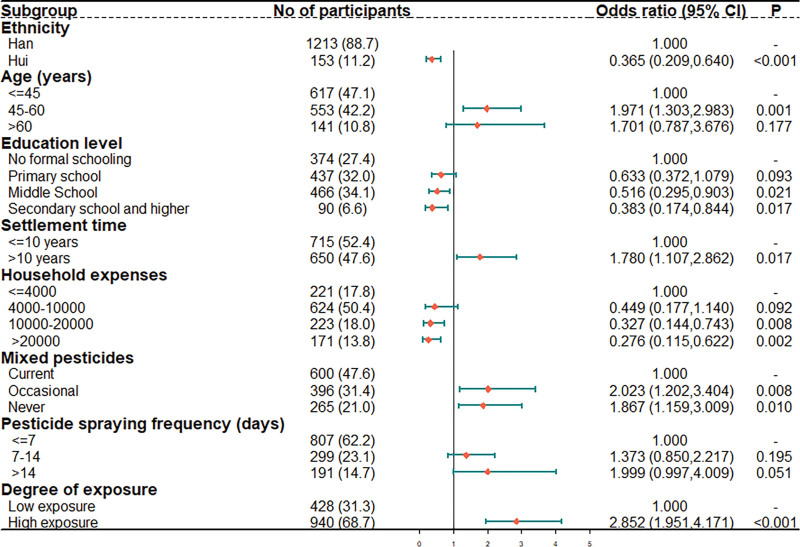
Forest plot demonstrating coronary heart disease hazard ratios from Binary logistic regression models and 95% CI. Risk factors include age over 45 years, Han ethnicity, low household expenses, high consumption of sauerkraut, 2 or more mixed pesticides, high pesticide spraying frequency, and a high degree of pesticide exposure.

Figure 1 displays the results of a logistic regression model on CHD that incorporated ethnicity, age, education level, settlement time, household expenses, mixed pesticide status, pesticide spraying frequency, and the degree of pesticide exposure. After adjusting for these CHD disease-related variables, a settlement time over 10 years (HR = 1.780, 95% CI [1.107, 2.862], *P* = .017), age between 45 and 65 (HR = 1.971, 95% CI [1.303, 2.983], *P* = .001), mixed pesticides (HR = 2.023, 95% CI [1.202, 3.404], *P* = .008) and high pesticide exposure (HR = 2.852, 95% CI [1.951, 4.171], *P* < .001) were associated with a significantly elevated incidence rate. Hui ethnicity (HR = 0.365, 95% CI [0.209, 0.640], *P* < .001), a high education level (HR = 0.516, 95% CI [0.295, 0.903], *P* = .021), and high household expenses (HR = 0.327, 95% CI [0.144, 0.743], *P* = .008) were protective factors against CHD.

### 3.3. DAG

**Figure 2.**
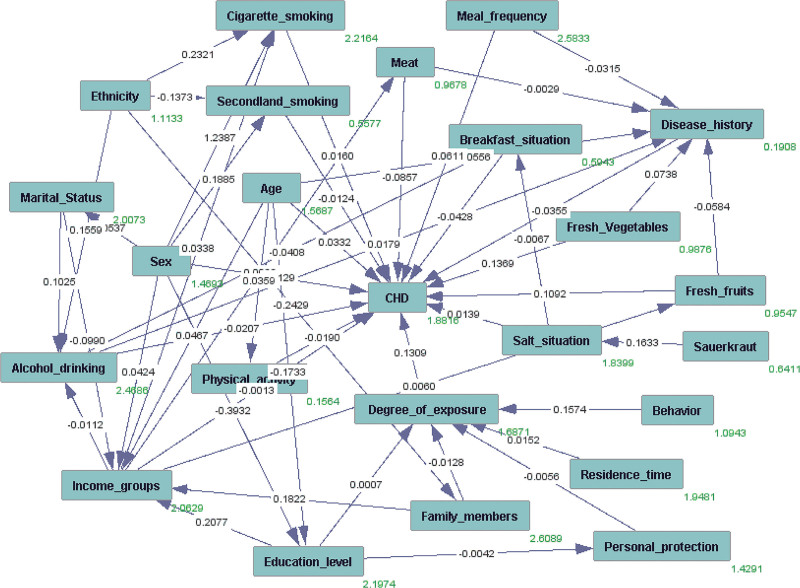
DAG is derived from literature and expert knowledge. Nodes represent variables and arrows represent causal associations between variables. The dark-colored nodes represent the factors affecting coronary heart disease collected from the questionnaire. Numbers between 2 variables represent partial regression coefficients; red numbers represent intercepts. CHD, coronary heart disease.

Figure 2 shows the resulting DAG according to references describing the empirically confirmed association between incident CHD and pesticide exposure. Although DAG can intuitively reflect the status and path of each variable in the occurrence of cardiovascular disease, it cannot intuitively reflect which pathway is meaningful. Therefore, it is necessary to test the DAG results. The results are shown in Table 2.

**Table 2:** CHD pathogenic pathway test results.

From	To	Value	SE	T	*P*	OR
Age	CHD	0.0332	0.0124	2.6696	.008	1.034
Sex	CHD	0.0129	0.0244	0.5313	.595	1.013
Income groups	CHD	–0.0013	0.0083	–0.1544	.877	0.999
Salt situation	CHD	0.0139	0.0111	1.2514	.211	1.014
Secondland smoking	CHD	–0.0124	0.0184	–.6762	.499	0.988
Alcohol drinking	CHD	–0.0207	0.0129	–1.6121	.107	0.980
Cigarette smoking	CHD	0.0160	0.0127	1.2536	.210	1.016
Physical activity	CHD	–0.0190	0.0249	–0.7633	.445	0.981
Breakfast situation	CHD	–0.0428	0.0231	–1.8541	.064	0.958
Fresh Vegetables	CHD	0.1369	0.0911	1.5033	.133	1.147
Meal frequency	CHD	0.0611	0.0210	2.9137	.004	1.063
Meat	CHD	–0.0857	0.0565	–1.5168	.130	0.918
Fresh fruits	CHD	0.1092	0.0479	2.2813	.023	1.115
Disease history	CHD	–0.0355	0.0229	–1.5486	.122	0.965
Degree of exposure	CHD	0.1309	0.0196	6.6921	<.001	1.140
Education level	Degree of exposure	0.0007	0.0143	0.0521	.9585	1.001
Personal protection	Degree of exposure	–0.0056	0.0169	–0.3304	.7411	0.994
Residence time	Degree of exposure	0.0152	0.0133	1.1416	.2538	1.015
Behavior	Degree of exposure	0.1574	0.0429	3.6708	.0003	1.170
Family members	Degree of exposure	–0.0128	0.0185	–0.693	.4884	0.987

The table shows the test results of the pathogenic pathways of CHD. The value represents regression coefficient, the sign represents positive or negative correlation. SE represents standard error; T represents statistic; OR represents odds ratio.

### 3.4. The DAG test results of the pathogenic pathways of CHD

Age was significantly associated with CHD in both regression models and DAG tests (the coefficient is 0.0332 and the OR is 1.034, *P* < .05). A similar trend occurred in the degree of pesticide exposure (the coefficient is 0.1309 and the OR is 1.141, *P* < .05). Meal frequency and consumption of fresh fruit were associated with a significantly elevated CHD incident rate in DAG tests (*P* < .05). Additionally, the results of DAGs showed that the factors of risk behavior behaviors during the spraying of pesticides such as smoking, eating, chatting, etc impact the effect of pesticide exposure. However, the factors of education level, settlement time, and mixed pesticide status were no significant association with CHD, which indicated that these are potential confounding factors by comparing the results with the regression analysis (Table 2).

## 4. Discussion

This study investigated the relationship between the degree of pesticide exposure and the development of CHD among greenhouse vegetable farmers. High pesticide exposure was independently associated with risk factors for CHD by both DAG and logistic regression analysis. After adjusting the additional potential influence of factors identified by the DAG, there was no significant association, such as the results in logistic regression: education level, settlement time, and mixed pesticide status. Specifically, age, meal frequency, and consumption of fresh fruit, by DAG analysis, were independent factors of CHD.

A DAG analysis allowed stronger inferences of causality, revealing a much more complex causal path.^[33]^ It refuted the association between the degree of pesticide exposure and CHD by several of the putative regression mediators. Thus, a DAG analysis provided no support for education level, settlement time, and mixed pesticide status since these variables were all found to be downstream of the degree of pesticide exposure or others. The DAG showed that the degree of pesticide exposure was the least common ancestor of CHD; education level, residence time, and mixed pesticide status are common ancestors of the degree of pesticide exposure, and hence they could appear as confounding factors that could bias regression analyses. A regression association between education level and CHD was estimated. Populations that had a high education level might be at a lower risk for pesticide exposure in agricultural practices, possibly because such people clearly understood the instruction manual and safety procedures included on the pesticide product labels.^[34]^ In addition, a settlement time of over 10 years and 2 or 3 kinds of pesticides mixed can increase the risk of pesticide exposure and indirectly affect the occurrence of CHD. According to research conducted among pesticide factory workers, the highest susceptibility for developing CHD occurs 10 to 19 years after pesticide exposure.^[35]^ According to Charles et al, the greatest association between exposure to chemicals such as pesticides, metals, and solvents and total mortality was found 15 years before death.^[12]^ In addition, the health effects of chemical pesticides may have particular synergistic effects due to the mixed-use of pesticides. A recent study in mice assessed the metabolic effects of chronic ingestion of a mixture of 6 common pesticides at acceptable intake levels. Consuming pesticide-contaminated chow resulted in metabolic disruption including increased body weight, greater adiposity, and glucose intolerance including weight gain, obesity and glucose tolerance in male mice, and increased fasting hyperglycemia and oxidative stress in female mice.^[36]^

The current study’s DAG analysis indicated that age, meal frequency, consumption of fresh fruit, and degree of pesticide exposure are common ancestors of CHD, which is itself an independent factor. Interpretively, respondents who had less pesticide exposure tended to have a lower risk of CHD in some settings of age, meal frequency, and consumption of fresh fruit. The typical routes of exposure to pesticides in farmers are absorption, inhalation, and ingestion. A prospective study found that long-term intake of fruit contaminated with pesticide residues was positively associated with a lower risk of CHD, with a 20% lower risk of CHD for a consumption < 1 serving/day compared to an intake of 4 or more servings/day.^[11]^ Consistent with our findings, a long-term intake of fruit contaminated with pesticide residues is an adverse factor in CHD (OR: 1.11; 95% CI 1.02–1.22). Nutrients and phytochemicals in fruits not only affect the self-inflammatory responses but also impact cellular redox processes and endothelial and tissue metabolic processes, but pesticide residues on fruits may hinder their effects, thereby increasing the risk of CHD.^[37]^ Both in vivo and ex vivo experiments have shown a few pesticides impact the cardiovascular system.^[12]^ Several common pesticides such as organophosphates (via effects neurologic functions by inhibiting acetyl-cholinesterase), organochlorine pesticides^[38,39]^ (via alteration of the ligand-gated ion channel to remain open), and pyrethroids^[40]^ (via modification of the kinetics of voltage-gated sodium channels leading to prolongation of the deactivation of sodium channels) have been confirmed to induce cellular oxidative stress and promote apoptosis, as well as playing important roles in the occurrence and progression of cardiovascular disease. Although a great number of greenhouse practitioners had a clear perception that pesticides were hazardous to their health, the use of PPE (i.e., boots, protective hats, gloves, protective masks, and thicker or impermeable clothes) worn during pesticide spraying was not a common practice and even appear some reckless behavior in some rural areas. Still, 45% of farmers engaged in unhealthy behavior during pesticide spraying, such as eating, drinking, smoking, and chatting. The reasons for such behavior may be due to the different age composition of the area (53% of farmers are over 45 years old), and the difference in education level (6.6% are high school education level or above), Such farmers usually have poor self-protection awareness and inadequately protect their health during pesticide spraying. These results point out that behavior is an essential route of exposure to pesticides that indirectly affect the occurrence of CHD.

DAG represents a considerable advance over standard logistic regression techniques. A possible explanation for this is that first, traditional mathematical–statistical methods in a complex setting may lead to a confounding bias rather than reducing it.^[41]^ In the conventional method of adjustment, a large number of confounders are used in stepwise variable selection methods, which may lead to unstable models and an increase in the risk of selecting the wrong covariates.^[42]^ Conversely, DAG provides a graphical and mathematically sound methodology to identify a suitable minimal confounder bias in epidemiologic studies. The backtracking algorithm empowers the identification of minimally sufficient variable sets, which are composed of the minimum amount of covariates needed to account for confounding, selection, overadjustment, and detection bias.^[43]^ Second, DAG can offer more information than logistic regression methods, especially for observational studies,^[44]^ analyzing and inferring both the strength and direction of the possible associations between the entire set of variables and tips improbable causal relationships.^[45,46]^ Nevertheless, caution must be exercised as causal inferences can only be supported if they are faithful and sufficiently causal.^[7]^

Limitations of our study must be noted. Due to the cross-sectional design of the study, a memory recall bias and selection biases could exist, especially for specific pesticide use and personal information recall, which might affect the precision of the results. As with other statistical methods, the use of DAG also depends on the data quality. The survey is in the form of a questionnaire, but may not fully capture the underlying structure. Furthermore, we binarized some variables, which inevitably led to some loss of information. Practically, the temporal relationship between variables cannot be determined because they can be measured simultaneously. The accuracy of a DAG is also compromised if the causal relationship between variables is distorted. While DAG is useful for conceptualizing causal relationships qualitatively, advanced quantitative causal models should be used for estimating causal effects.

## 5. Conclusion

The associations between CHD and pesticide exposure were found in binary logistic regression analyses and DAG analyses. Several of the putative regression mediators became no longer significant after adjustment for a minimally sufficient set of covariates extracted from a literature-based DAG. Ultimately, the DAG analysis indicated age, meal frequency, consumption of fresh fruit, and degree of pesticide exposure are the common ancestors of CHD, which is also an independent factor. Thus, effective measures should be adopted by the local government such as protective gear to limit occupational pesticide exposure, intervention in lifestyle and agricultural activities among farmers, and reducing the increased risk of developing CHD. DAG, which are visual representations of causality in scientific discussions, are increasingly used in modern epidemiology. Using a DAG could potentially reduce the likelihood of a poor adjustment caused by collinearity, intermediates, or colliders.^[45]^ DAG can be a preferable improvement over traditional regression methods, especially in complex research questions and causal inference in observational studies.

## Acknowledgments

This is a short text to acknowledge the contributions of specific colleagues, institutions, or agencies that aided the efforts of the authors.

## Author contributions

**Data curation:** HongHui Li, Yue Zhang.

**Investigation:** HongHui Li.

**Software:** Jiangping Li.

**Writing – original draft:** Cheng Zheng.

**Writing – review & editing:** Huifang Yang, Jiangping Li.

## Supplementary Material


