# Supplementary material for: The directed acyclic graph helped identify confounders in the association between coronary heart disease and pesticide exposure among greenhouse vegetable farmers
Source: Medicine (Baltimore). 2023 Sep 22;102(38):e35073. doi: 10.1097/MD.0000000000035073 (PMC10519556; doi:10.1097/MD.0000000000035073)
Supplement: Supplementary file 1 [file medi-102-e35073-s001.docx]

| **Supplementary Table 1. Specified scores for combinations of PPE use** | |
| --- | --- |
| Type of PPE | Scores |
| PPE-0 | 1.0 |
| PPE-1 | 0.8 |
| PPE-2 | 0.7 |
| PPE-3 | 0.6 |
| PPE-1 & PPE-2 | 0.5 |
| PPE-1 & PPE-3 | 0.4 |
| PPE-2 & PPE-3 | 0.3 |
| PPE-1 & PPE-2 & PPE-3 | 0.1 |
| Abbreviation: Personal protective equipment, PPE; https://doi.org/10.1371/journal.pone.0209566.t001 | |
